# Evolution and Disease

**Published:** 1890-05

**Authors:** 


### REVIEW DEPARTMENT.

EVOLUTION AND DISEASE. BY BLAND SUTTON. Pp. xiii, 285, with 136 illustrations. London : Walter Scott, 1890.

So ephemeral is much of contemporary medical, veterinary and biological literature that seldom can the reviewer bring that amount of enthusiasm to his task which grows out of the reflection that the book may not have been forgotten before his review can be written and read. It is a pleasure to review the work before us. It is emphatically a work which cannot be opened without being perused ; which cannot be so perused without evoking thought, and which few, if any, can lay down without having learned from it some new fact or novel suggestion. It occupies about the same relation to comparative pathology which Huxley's "Anatomy of the Cray-fish" bears to zootomy. It can be read by non-specialists, without being, as are most popular works, unprofitable to the specialist, for it successfully covers a range of subjects so large as seldom comes under the ken of a single observer.

Starting with the canon—which is indeed a *sequitur* of the difficulty which medical writers find in defining disease, or determining when health ends and illness begins—that pathology is truly a department of biology, he proceeds almost in a colloquial way to illustrate this by citing the numerous cases where what is a normal structure in one animal becomes an abnormal or pathological one by appearing in another. A most happy illustration of this is the development of hair on the inner mucous surface of the cheeks in human infants, such being comparable to the normal growths of hair in the same situation in rodents.

Mr. Sutton is one of those industrious observers and collators of facts whom nothing pertinent seems to escape. In immediate connection with the subject just mentioned we may refer, as an illustration of his polyhistoric knowledge, to his reference to the gentleman surprised at his dinner by finding a cyst containing sebaceous and hirsute contents in a beef tongue.

The peculiarities of feather growth in birds, the accidental development and subsequent permanent retention of monstrous characters, such as the fifth claw in the Dorking fowl, are fully considered. The interesting subject of imperfect sexual development, as shown in the male plumage sometimes acquired by female birds, is explained in a very satisfactory way.

There is scarcely a branch of morphology, embryology, phylogeny, heredity, cellular pathology or physiology which can even remotely be brought in relation to animal diseases that is not ably and suggestively handled. Most fascinating is the account of the contest for existence between leucocytes and bacilli in the interior of the microscopic water crustacean *Daphne*. To veterinarians the study of the last chapters, in which the variabilty in clinical manifestations of one and the same disease process in different animals and man, is traced to its proper course is commended. It is one of the most philosophical disquisitions on comparative pathology we have seen, and is alone worth obtaining the work for. To give an abstract of these interesting chapters is impossible, for the simple reason that the author has himself condensed his information into the smallest possible space, thus rendering it equally concise, full and clear.

The book is fully illustrated; many of the figures are after original drawings of the author, which appeared for the first time in the JOURNAL OF COMPARATIVE MEDICINE AND SURGERY.

It may seem carping at trifles, but as English critics in the *Lancet* and *British Medical Journal* have repeatedly referred to the *American* vice of describing the dimensions of tumors and other objects by saying "of the size of a nickel, or a quarter, or a peanut," we would call attention to a "four-penny piece" (p. 135), given as the size of a teratological growth in a man's heart. This vague statement, however, Dr. Ogle, from whom the case is cited, is responsible for.

All in all, we must congratulate the publishers of the Cotemporary Science Series for their wise selection of Mr. Sutton's book.

